# Multi-strain probiotics during pregnancy in women with obesity influence infant gut microbiome development: results from a randomized, double-blind placebo-controlled study

**DOI:** 10.1080/19490976.2024.2337968

**Published:** 2024-04-09

**Authors:** Sofie Ingdam Halkjær, Malene Refslund Danielsen, Victoria E. de Knegt, Lee O’Brien Andersen, Christen Rune Stensvold, Henrik Vedel Nielsen, Hengameh Chloé Mirsepasi-Lauridsen, Karen Angeliki Krogfelt, Dina Cortes, Andreas Munk Petersen

**Affiliations:** a Gastrounit,Medical Division, Copenhagen University Hospital Amager and Hvidovre, Hvidovre, Denmark; b Department of Pediatrics and Adolescent Medicine, Copenhagen University Hospital Amager and Hvidovre, Hvidovre, Denmark; c Department of Bacteria, Parasites and Fungi, Statens Serum Institut, Copenhagen, Denmark; d Department of Science and Environment, Roskilde University, Roskilde, Denmark; e Department of Clinical Medicine, Faculty of Health and Medical Sciences, University of Copenhagen, Copenhagen, Denmark; f Department of Clinical Microbiology, Copenhagen University Hospital Amager and Hvidovre, Copenhagen, Denmark

**Keywords:** Obesity, microbiome, microbiota, probiotics, pregnancy, newborn, infant

## Abstract

Probiotics have been described to influence host health and prevent the risk of obesity by gut microbiome (GM) modulation. In a randomized double-blinded placebo-controlled feasibility study, we investigated whether Vivomixx® multi-strain probiotics administered to 50 women with obesity during pregnancy altered the GM composition and perinatal health outcomes of their infants up to 9 months after birth. The mothers and infants were followed up with four visits after birth: at 3 d, and at 3, 6, and 9 months after delivery. The infants were monitored by anthropometric measurements, fecal sample analysis, and questionnaires regarding health and diet.

The study setup after birth was feasible, and the women and infants were willing to participate in additional study visits and collection of fecal samples during the 9-month follow-up. In total, 47 newborns were included for microbiome analysis.

Maternal prenatal Vivomixx® administration did not alter infant GM diversity nor differential abundance, and the probiotic strains were not vertically transferred. However, the infant GM exhibited a decreased prevalence of the obesity-associated genera, *Collinsella*, in the probiotic group and of the metabolic health-associated *Akkermansia* in the placebo group, indicating that indirect community-scale effects of Vivomixx® on the GM of the mothers could be transferred to the infant.

Moreover, 3 d after birth, the GM of the infant was influenced by mode of delivery and antibiotics administered during birth. Vaginally delivered infants had increased diversity and relative abundance of the metabolic health-associated *Bifidobacterium* and *Bacteroides* while having a decreased relative abundance of *Enterococcus* compared with infants delivered by cesarean section. Maternal antibiotic administration during birth resulted in a decreased relative abundance of *Bifidobacterium*in the GM of the infants. In conclusion, this study observed potential effects on obesity-associated infant GM after maternal probiotic supplementation.

## Introduction

Obesity among children and adolescents is a global health issue with increasing trends.^1^ This condition is associated with mental health complications, several sequelae (including type-2 diabetes, cardiovascular diseases and cancerous diseases), and increased health-care expenses.^2,3^ Obesity thus constitutes a severe health concern for affected individuals as well as capacity-related and economical concerns for the health-care system.

Maternal obesity and excessive gestational weight gain (GWG) are associated with increased risk for offspring obesity, and these associations are not fully explained by genetic and lifestyle factors. Evidence suggests that the maternal gut microbiome (GM) impacts the early infant GM.^4^ Therefore, it has been hypothesized that the GM may be a mechanism to explain the transgenerational transmission of obesity risk.^5^ This hypothesis is supported by research showing that maternal GWG and Body Mass Index (BMI) influence maternal and infant GM.^6^

The early colonization of the infant gut is characterized by an initial low bacterial diversity, which increases over time from birth as the gut becomes increasingly colonized.^7^ As the infant is exposed to the maternal microbiome from the moment of birth (through vaginal fluids and fecal matter during vaginal delivery, skin-to-skin contact, or breastfeeding), the early GM of the infant is likely to bear resemblance to that of the mothers.^8^ Early-life colonization of the gut with microorganisms is thus also highly influenced by factors such as mode of delivery (vaginal or cesarean section), feeding method (breastfeeding or formula feeding), and exposure to antibiotics^9–11^ High exposure to the maternal microbiome as vaginal delivery, breastfeeding until 6 months of age, and minimal exposure to antibiotics are therefore considered optimal colonization conditions for developing a healthy gut.^10^ However, if the mother suffers from obesity, the resulting obesity-associated composition of the maternal GM can be transferred to the neonate, resulting in an increased risk of developing obesity later in life.^5^ In an attempt to promote the transfer of bacteria associated with health benefits from mother to child, some studies successfully administered probiotics during pregnancy, under the hypothesis that this might help reduce the risk of obesity and other metabolic diseases later in life.^12,13^ Luoto *et al*. ^12^ administered probiotic *Lacticaseibacillus rhamnosus* GG (formerly known as *Lactobacillus rhamnosus* GG) to obese mothers both during pregnancy and after birth and found promising results in restraining weight gain during the first years of life; the effect, however, diminished over time from the age of 2 y. This highlights both the intricate relationship between the GM and obesity but also the great potential of exploiting probiotics as modulators of the GM and thereby health and disease.

Generally, the GM of humans consists mainly of bacteria from the two phyla *Bacteroidetes* and *Firmicutes*; however, members of the phyla *Actinobacteria, Proteobacteria*, and *Verrucomicrobia* are also commonly found.^14^ Compositional changes in the GM have been found to affect energy balance, glucose metabolism, and inflammation, all of which are commonly observed in obesity and metabolic disorders such as insulin resistance and type-2 diabetes.^15^ Across the literature, the GM of human individuals and animal models with obesity are characterized by low bacterial richness and diversity as well as altered metabolic functions of the GM, as opposed to the high richness and diversity associated with the microbiome of healthy and lean counterparts.^16^ Studies have further suggested a decreased relative abundance of *Bacteroidetes* and an increased relative abundance of *Firmicutes* in ‘obese microbiomes’ compared with ‘lean microbiomes’.^17,18^ However, these taxonomic characteristics of the composition of ‘obese microbiomes’ have not been consistently observed across studies. This suggests the existence of either methodic challenges and/or a more complex relationship between microbiome and obesity beyond the mere imbalance in the abundance of commensal phyla^19–21^ The underlying mechanisms of the relationship between GM and obesity are not fully elucidated. However, the significant role of the GM in the disposal, development, and preservation of obesity is becoming increasingly evident and indicates the potential for new treatments with life biotherapeutic products.^22^ Consequently, supplementation with live bacteria with potential health benefits to the host, known as probiotics, is increasingly used to increase GM diversity, contributing to the improved metabolic functions of the GM.^23^

In our previous randomized controlled trial (RCT), Vivomixx® multi-strain probiotic administration during pregnancy for women with obesity increased GM diversity significantly.^24^ In this study, we aimed to evaluate the extent to which this effect is reflected in the GM composition of the infants of these women from 3 d until 9 months after birth. We also evaluated whether maternal probiotic treatment affected infant body weight development, which could indicate an effect on obesity predisposition.

## Material and methods

### Study design

This feasibility RCT was carried out at Copenhagen University Hospital Hvidovre, Denmark, from February 2015 to January 2018 and included 50 pregnant women with obesity randomly assigned to treatment groups 1:1 to receive capsules containing Vivomixx® (Visbiome® in North America, DeSimone Formulation® in Asia) or placebo from gestational weeks 14–20 until delivery. The Vivomixx® multi-strain probiotic holds a daily intake of a concentration of 450 billion CFU/day and consists of eight different bacterial strains, including *Streptococcus thermophilus* NCIMB 30438, *Bifidobacterium breve* NCIMB 30441, *Bifidobacterium lactis* NCIMB 30435 (formerly known as *B. longum*), *Bifidobacterium lactis* NCIMB 30436 (formerly known as *B. infantis), Lactobacillus acidophilus NCIMB 30442, Lactobacillus plantarum NCIMB 30437, Lactobacillus paracasei NCIMB 30439*, and *Lactobacillus helveticus NCIMB 30440* (formerly known as *L. delbrueckii ssp. bulgaricus)*. The Vivomixx® formulation was chosen based on previously interesting results described in our published protocol.^25^ The placebo treatment capsules contained microcrystalline cellulose, magnesium stearate, and silicon dioxide. The pregnant women were randomly assigned 1:1 to receive probiotic (Vivomixx®) or placebo capsules and included by consecutive numbers. Randomization was done in blocks of four, and both probiotic and placebo capsules were identical in appearance and packaging. All participants and contributors in the study were blinded to the interventions. The randomization key was revealed to the researchers only when all participants had completed the 9-months follow-up visit and data analysis was complete.

The women and their newborns were in this part of the study followed until 9 months after delivery, including four visits after birth: 3 d (1–3 d) after delivery, 3 months after delivery, 6 months after delivery, and finally 9 months after delivery according to the study protocol.^25^ These visits included a collection of fecal samples, anthropometry measurements, and administration of general health questionnaires (with a view to collecting data on allergy, atopic dermatitis, colic or others, diet [breast milk, formula milk, and introduction of solid food] and concomitant medications [including antibiotics and probiotics] from the participants and their infants).

The weight development of the infants was compared to the international growth charts “Birth to 24 Months: Weight-for-age Percentiles”, for girls and boys, respectively, created by The World Health Organization (WHO) from 2006.^26^ For identification of children with adverse weight development, WHO recommends considering weight values 2 standard deviations below and above the median corresponding to the 2.3rd and 97.7th percentiles.^27^ The infants were thus divided into three groups: below 2 SD of the median (defined as below the 2.3rd percentile), normal (defined as in between the 2.3rd and 97.7th percentiles) and above 2 SD of the median (defined as above the 97.7th percentile).

### Participants

The pregnant women and their newborns were recruited after the following inclusion criteria: above 18 y of age, a BMI of 30–35 kg/m^2^ (calculated using pre-pregnancy weight data), primiparous singleton pregnancy, Danish language (spoken and written), normal ultrasound scan of the fetus at gestational age 12–14 weeks, and consent to an oral-glucose-tolerance test at gestational age 14–20 weeks. Exclusion criteria included a gestational age >20 weeks at recruitment time, pregestational diabetes or other severe diseases, multiple pregnancy, previous bariatric surgery, ingestion of probiotics within 1 month before inclusion, and alcohol or drug abuse. The study protocol is published in detail elsewhere.^25^

Exclusion criteria for this study and data analysis included infants born before term. Moreover, fecal samples from infants that after birth received either probiotics or antibiotics during the 9-month follow-up were removed from the main dataset to reduce variance and the potential effect of this probiotic and antibiotic supplementation on the GM of the infants.

### Outcomes

Infant outcomes included anthropometry measurements, and general health questionnaires, including data on allergy, atopic dermatitis, colic or others, diet (breast milk, formula milk, and introduction of solid food) and concomitant medications, including antibiotics and probiotics. In addition, fecal samples were collected to compare differences in gut microbiome in the two groups (infants of mothers treated with probiotics or placebo during pregnancy). All outcomes were compared at 3 d after delivery, and at 3, 6 and 9 months after delivery as described in the study protocol.^25^

### Ethics

The study was approved by the Danish Data Protection Agency (AHH-2015-001), and permission for human experiments and recruitment of participants was obtained from the Scientific Ethics Committee for Copenhagen Regional Hospitals, Denmark (Permission no.: H-2-2014-076) version 2.1, December 5, 2014.

The study was performed in accordance with the Revised Declaration of Helsinki. The study was registered at www.clinicaltrials.gov as NCT02508844. All participants provided written informed consent to participate after verbal and written information was given. For each included newborn, informed written consent was obtained from both parents. Participants were informed that they could withdraw from the study at any time.

### Fecal microbiome DNA extraction and sequencing

DNA extraction, library development, and sequencing of the fecal samples and a positive control of Vivomixx® were accomplished as described in the study protocol.^25^ Fecal samples were collected by the mothers from the infants at home, sent by mail, and then frozen at −80°C. DNA extraction was performed using the PowerSoil DNA Isolation Kit (QIAGEN, Hilden, Germany). The DNA was amplified using a two-step polymerase chain reaction (amplification and adaptor PCR, respectively) and a modified version of the universal prokaryotic 341F/806 R primers targeting the V3-V4 hyper-variable regions of the 16S rRNA region.^28^ The modification of the primers included three additional nucleotides attached in the 5’ end of the forward primer (ACTCCTAYGGGRBGCASCAG, 341F3) and five additional nucleotides attached in the 5’ end of the reverse primer (AGCGTGGACTACNNGGGTATCTAAT, 806R5). DNA concentration was quantified using Quant-IT™ dsDNA High Sensitive Assay Kit (Thermofisher Scientific, Waltham, Massachusetts, USA) and pooled equimolarly. Pooled amplicon libraries were cleaned for DNA fragments of undesirable length using Agencourt AMPure XP beads (Beckman Coulter Brea, California, USA), removing fragments below 300 bp and fragments above 1000 bp using a ratio of 10:24 and 10:16 of pooled amplicon libraries to AMPure beads, respectively. The purified amplicons were sequenced on the Illumina MiSeq desktop sequencer (Illumina Inc., San Diego, California, USA) with the 500 rxn MiSeq Reagent Kit V2 in a 2 × 250nt setup. A maximum of 64 samples were sequenced in a single run. To ensure correct taxonomical detection of the Vivomixx® probiotic strains after sequencing of the fecal samples, a capsule of Vivomixx® was sequenced as a positive control.

### Sequencing output data processing

The sequencing output was quality trimmed, tested for chimeras and taxonomically mapped using BION (http://box.com/bion), a *k*-mer based mapping software developed by the Danish Genome Institute (Aarhus, Denmark), the Danish Veterinary Institute (Copenhagen, Denmark) and Statens Serum Institute (Copenhagen, Denmark). The software accepts raw sequences and performs the following functions: primer sequence extraction, cleaning, pair mate joining, length and quality trimming, sequence unification, de-replication, chimera filtering, clustering, reference similarity, similarity profiling and produces taxonomy profile tables to all taxonomic levels. Query sequences were compared to the 358–792 bp region (corresponding to the 16S rRNA V3-V4 gene position) of the Ribosomal Database Project, RDP (Release 11, update 5, September 30, 2016).^29^

### Statistics analysis

All statistical analyzes and plotting were preformed using R-studio version 4.1.2.^30^ Rhea, a publicly available bioinformatic pipeline written in R-language, was used to analyze microbial profiles.^31^ The pipeline includes normalization using rarefaction curves, alpha and beta diversity calculation, taxonomic binning, serial-group statistical testing and correlation analysis. Additional analysis was done using the phyloseq package (phyloseq: An R package for reproducible interactive analysis and graphics of microbiome census data).^32^ Alpha diversity was tested using Wilcoxon rank sum tests, Beta diversity was tested with PERMANOVA tests and differential abundance was evaluated using DESeq2.^33^ All tests comparing timepoints were done as paired tests. Statistical correction was performed using Benjamini & Hochberg False discovery rate-corrected *p* values.

## Results

### Infants and fecal sample sequencing

**Figure 1.**
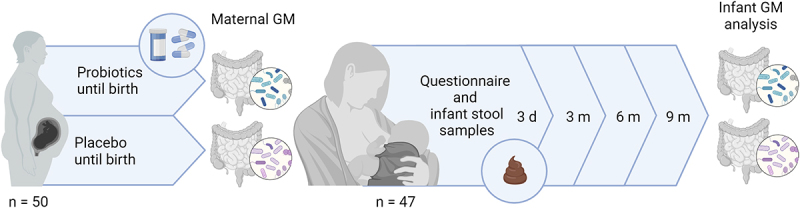
Study overview. A total of 50 pregnant women with obesity received either Vivomixx® probiotic or a placebo treatment from 14–20 weeks of gestation until birth. Administration of probiotics significantly increased the diversity of the GM compared to the placebo group as published elsewhere.^34^ for the remainder of the study period, 47 infants were included with physical examination and health questionaries, besides having fecal samples collected 3 d, 3 months, 6 months, and 9 months after birth. This was done to evaluate whether the maternal prenatally supplemented probiotics influenced infant GM diversity and relative abundance. This figure was created with BioRender.com.

In total, 47 newborns were included for microbiome analysis (24 from mothers given probiotics, including 14 born by vaginal delivery [VD] and 10 born by cesarean section [CD] and 23 from mothers given placebo including 19 VD and four CD) (Figure 1). Each newborn provided at least one fecal sample profiled at adequate sequencing depth. A total of 140 fecal samples were analyzed (Table S1) and included number of total reads: 12,060,782, mapping %: On average, samples mapped 99.6% (ranging from 94.38–100.01%) corresponding to 12,013,397 out of 12,060,782 reads. In total 710 unique OTUs were found and were distributed on 10 phyla, 275 genus and 633 species. Relevant data on the characteristics of the infants are presented in Table 1.

**Table 1.** Characteristics of the infants of mothers treated with probiotics or placebo during pregnancy included in the study.

	Probiotics (*n*=24)		Placebo (*n*=23)		Total
	N, (%)	mean	N, (%)	mean	n
Male sex	12 (50%)		12 (52%)		24
Cesarean delivery	10 (42%)		4 (17%)		14
Vaginal delivery	14 (58%)		19 (83%)		33
Birth weight (g)		3414		3638	
Weight at 3 d (g)		3222		3392	
Weight at 3 months (g)		6121		6380	
Weight at 6 months (g)		7995		8352	
Weight at 9 months (g)		9690		9852	
Gestation (d)		274		279	
Premature infants	4 (17%)		0 (0%)		4
Food just after birth, breastmilk exclusively	19 (79%)		18 (78%)		37
Food just after birth, formula exclusively	2 (8%)		0 (0%)		2
Food just after birth, combination	3 (13%)		5 (22%)		8
Food at 3 months, breastmilk exclusively	10 (42%)		17 (74%)		27
Food at 3 months, formula exclusively	7 (29%)		2 (9%)		9
Food at 3 months, combination	7 (29%)		4 (17%)		11
Solid food introduced after 3 months but before 6 months	22 (92%)		19 (83%)		41
Solid food introduced after 6 months	2 (8%)		4 (17%)		6
Food at 6 months, breastmilk exclusively	2 (8%)		6 (26%)		8
Food at 6 months, formula exclusively	2 (8%)		1 (4%)		3
Food at 6 months, combination (breastmilk + formula)	0 (0%)		1 (4%)		1
Food at 6 months, breastmilk + solid foods	5 (21%)		8 (35%)		13
Food at 6 months, formula + solid foods	12 (50%)		5 (22%)		17
Food at 6 months, breastmilk + formula + solid foods	2 (8%)		3 (13%)		5
Food at 9 months, breastmilk + formula	0 (0%)		1 (4%)		1
Food at 9 months, breastmilk + solid foods	5 (21%)		11 (48%)		16
Food at 9 months, formula + solid foods	14 (58%)		8 (35%)		22
Food at 9 months, breastmilk + formula + solid foods	3 (13%)		4 (17%)		7
Atopic dermatitis	2 (8%)		2 (9%)		4
Colic	1 (4%)		1 (4%)		2
Allergy	0 (0%)		1 (4%)		1
Infant antibiotic intake	1 (4%)		1 (4%)		2
Mothers who got antibiotics during pregnancy	6 (25%)		5 (22%)		11
Maternal antibiotics during the delivery of the child	18 (75%)		14 (61%)		32
Maternal antibiotics after the infant was born between 3 d and 9 months after birth	10 (42%)		10 (43%)		20

Notes: This table excludes the three participants who failed to complete all the after-birth visits but includes the infants that were prematurely born, receiving probiotics and antibiotics, respectively.

Abbreviations: G: gram; n: number.

Fecal samples from infants that after birth received either probiotics or antibiotics during the 9-month follow-up were removed from the main dataset to reduce variance and the potential effect of this probiotic and antibiotic supplementation on the GM of the infants. A total of five infants (three from the probiotic group and two from the placebo group) were given probiotics as a supplement by the mother. Two infants were administered antibiotics during a period of illness. The main dataset included fecal samples from newborns born at term who had neither been given probiotics nor antibiotics during the 9-month follow-up.

### Alpha diversity increased over time and was unaffected by maternal prenatal probiotic treatment

**Figure 2.**
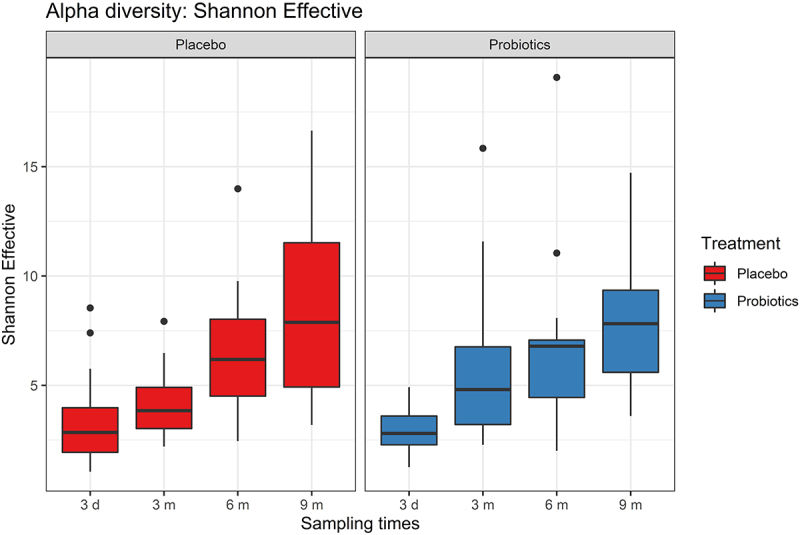
Alpha diversity (Shannon Effective) over time after birth (3 days [3d] 3 months [3 m], 6 months [6 m], and 9 months [9 m]) for the two groups; mothers treated with placebo (red) or probiotics (blue). There was a significant increase (*p* < 0.05) in alpha diversity over time for both groups, but the two groups were not significantly different from each other at any of the specific time points (Wilcoxon rank sum tests).

A significant increase (*p* < 0.005) in alpha diversity from the time of birth until 9 months after birth was observed in both treatment groups (Figure 2). This tendency applied to all alpha diversity metrics tested: Richness (exact, normalized, and effective), Shannon (Index and Effective), Simpson (Index and Effective), and Evenness (Table S2 and Table S3 and Fig. S1). Probiotic or placebo treatment of the mothers during pregnancy did not contribute to a significant difference in alpha diversity of infant GM, when comparing the two treatment groups at the same sampling times; 3 d, 3 months, 6 months, and 9 months, respectively (Figure 2). This applied to all alpha diversity metrics tested (Table S4 and Fig. S1).

### Significant change in infant gut beta diversity over time from birth, with maternal prenatal probiotic administration not affecting infant gut microbiota beta diversity

**Figure 3.**
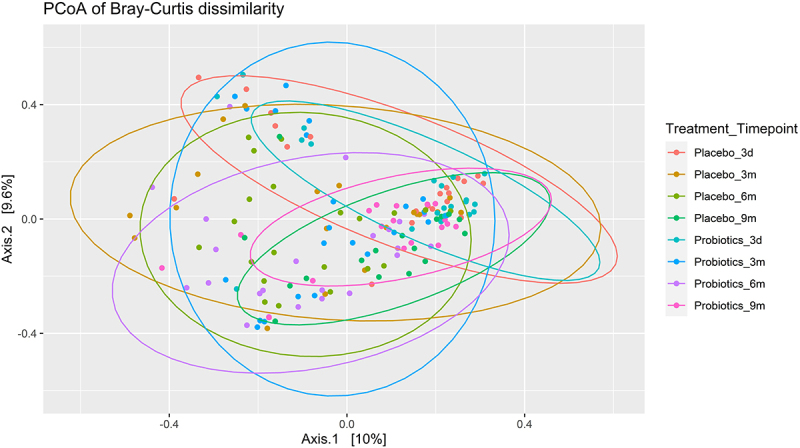
Principal coordinate analysis (PCoA) on the Bray-Curtis dissimilarity of infants whose mothers were treated with probiotic or placebo group during pregnancy. Infants were followed with four visits (3 days [3d], 3 months [3 m], 6 months [6 m], and 9 months [9 m] after birth, respectively).

When considering the Bray-Curtis dissimilarity analysis of the probiotic and placebo groups by principal coordinate analysis (PCoA), there were no significant differences between treatment groups at each time point (Figure 3). This was confirmed by PERMANOVA analysis, which revealed no significant difference in Bray-Curtis dissimilarity between any of the treatment groups. PCoA plots for each individual timepoint are available in the supplementary material (Fig. S2). When considering the difference across sampling times, there was, however, a significant change in Bray-Curtis dissimilarity over time from birth to 9 months of age for both treatment groups. These results are further supported by the weighted UniFrac (Fig. S3).

### No differences between infant GM relative abundance profiles in the probiotic and placebo groups from 3 d to 9 months of age

The differential taxonomic abundance between the two treatment groups were tested at phylum level, with no significant differences observed for any phylum at any sampling point. At phylum level, the infants in the two treatment groups were thus highly similar and displayed the same over-time patterns in phylum composition (Fig. S4), with no significant differences between the two groups. This tendency could be extrapolated to genus level, where no genera were found to differ significantly in abundance between the two treatment groups at any of the four sampling points.

### The relative abundances of species included in the probiotics were similar in the two treatment groups

**Figure 4.**
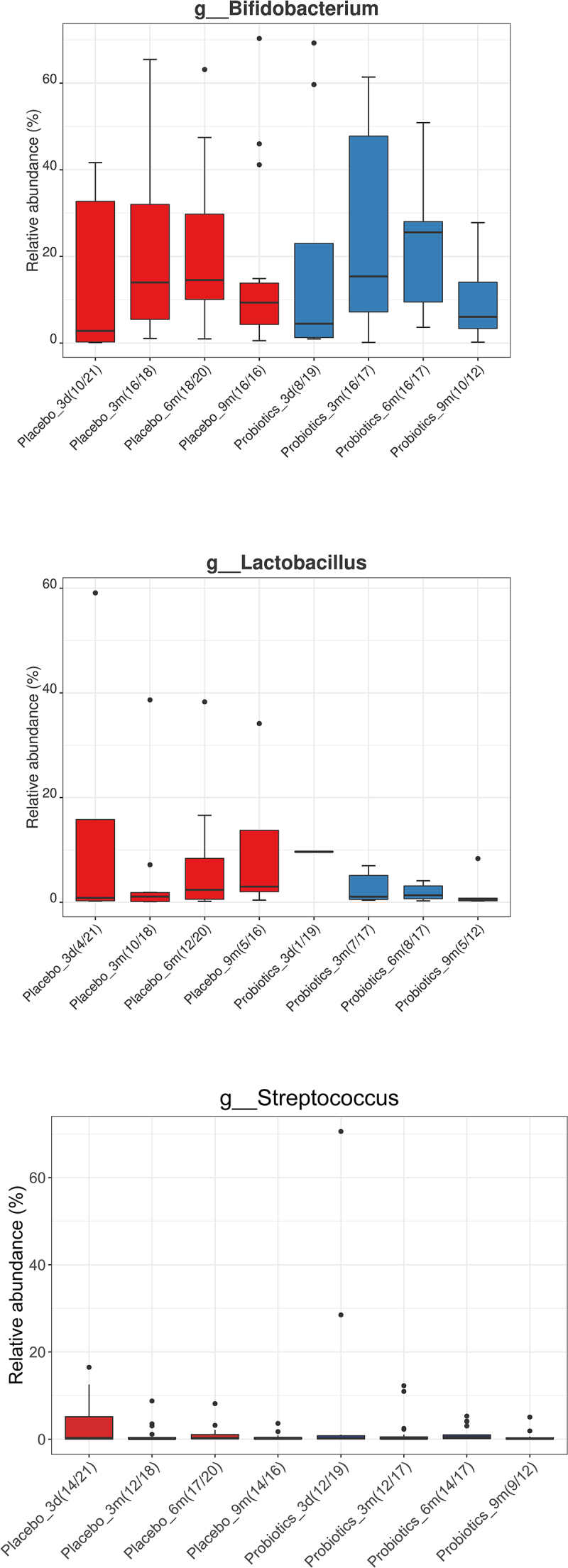
Relative abundance (%) of the genera *Bifidobacterium*, *Lactobacillus*, and *Streptococcus* respectively, for all time points (three days [3d], three months [3 m], six months [6 m], and nine months [9 m]) and for both; mothers treated with placebo (red) or probiotics (blue) during pregnancy. There were no significant differences between the groups at any of the specific time points.

The Vivomixx® probiotics administered to the mothers during pregnancy contained *Bifidobacterium* spp., *Lactobacillus* spp. and *Streptococcus salivarius*. When considering the relative abundance of *Bifidobacterium* spp., *Lactobacillus* spp., and *Streptococcus* spp. in the infants’ fecal samples, there was, however, no significant difference in the relative abundance between the probiotic and placebo treatment groups at any of the sampling times up to 9 months of age (Figure 4).

### No effects of maternal prenatal probiotic administration on infant weight development and prevalence of atopic dermatitis, colic, and allergy during the first 9 months of life

**Figure 5.**
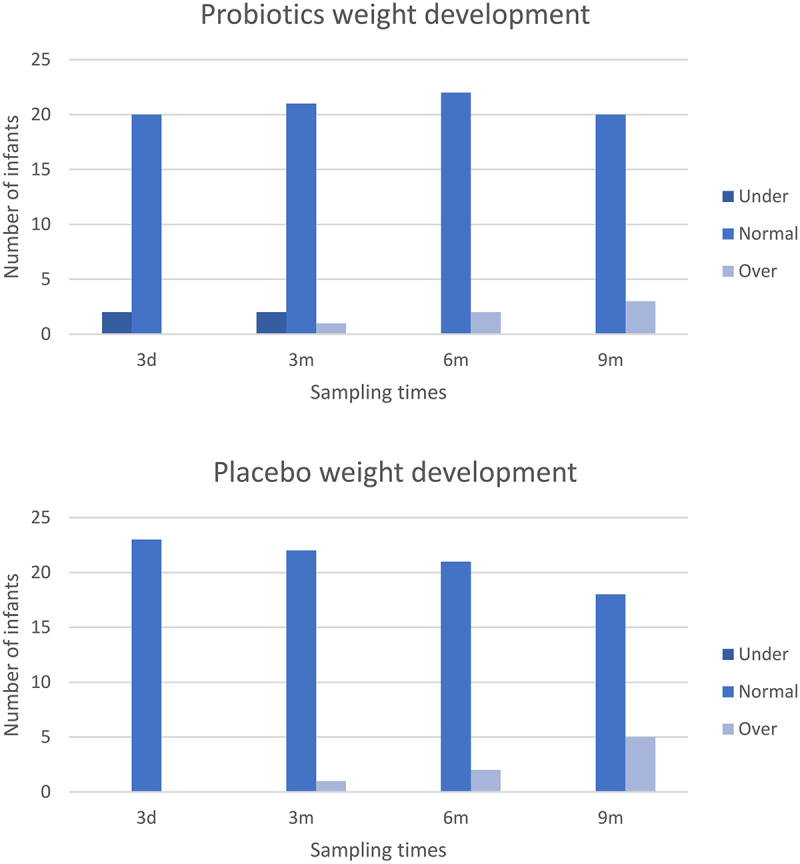
Infant weight development over time in infants from mothers treated with probiotics or placebo. The charts display the number of infants with a body weight either under 2 SD of the median (dark blue), normal (blue) or 2 SD over the median (light blue) for the specific age group (3 days [3d], 3 months [3 m], 6 months [6 m] or 9 months [9 m] of age, respectively) according to WHO standards.

The weight development of the infants was generally within the WHO standards, except for a few of individuals from the probiotic group. A body weight below two standard deviations (SD) of the WHO median was only observed at the first two sampling times (3 d and 3 months postnatally) (Figure 5). However, the number of infants with a body weight above 2 SD of the median increased over the last three sampling points (3 months, 6 months, and 9 months) (Figure 5). No difference between the two groups was found regarding the number of infants with a weight above 2 SD of the median.

Maternal prenatal Vivomixx® supplementation did not result in differences in prevalence of any diseases, including atopic dermatitis, colic, or allergy (Table 1).

### Mode of delivery: Short-term effect on diversity and relative abundance of Bacteroides, Bifidobacterium, and Enterococcus

**Figure 6.**
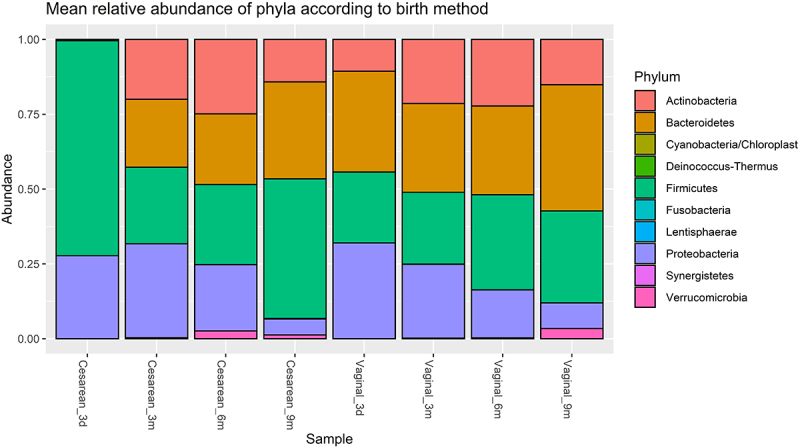
Mean relative abundance at phylum level according to delivery mode. Three days after birth (3d), the relative abundance of Firmicutes had significantly increased in the infants delivered by cesarean section (“Sectio”) compared with those born by vaginal delivery (“Vaginal”) (*p* = 0.006), and Bacteroidetes was significantly increased in infants born by vaginal delivery when these were compared with infants delivered by cesarean section (*p* = 0.0005). No significant differences were observed for the remaining time points; 3 months (3 m), 6 months (6 m), or 9 months (9 m).

A short-term effect of delivery mode was observed on alpha and beta diversity 3 d postnatally when comparing infants exposed to VD or CD. For alpha diversity, VD infants had a significantly higher Shannon Effective (*p* = 0.01) 3 d after birth compared with CD infants (Fig. S5). The same tendency was observed for beta diversity, with a significantly different Bray-Curtis dissimilarity 3 d after birth (*p* = 0.008) (Fig. S6). This short-term effect of delivery mode on infant GM was reflected in the differential abundance. The differences were especially pronounced at phylum level, where 3 d after birth, the GM of CD infants was constituted mainly by *Firmicutes* compared with VD infants, whose microbiomes were more diverse (Figure 6). The differential abundance of *Firmicutes* was thus significantly increased in CD infants compared with VD infants (*p* = 0.006), whereas *Bacteroidetes* was significantly increased in the VD infants compared with CD infants (*p* = 0.0005). This was further reflected at genus level 3 d after birth, where VD infants had a significantly higher relative abundance of *Bacteroides* (*p* = 0.03) and *Bifidobacterium* (*p* = 0.03) compared with the GM of CD infants, whereas *Enterococcus* (*p* = 0.03) was significantly increased in CD infants. This difference, however, quickly diminished, and at the remaining sampling points up to 9 months of age, no significant differences were observed at neither phylum nor genus level.

### Collinsella and Akkermansia findings

**Figure 7.**
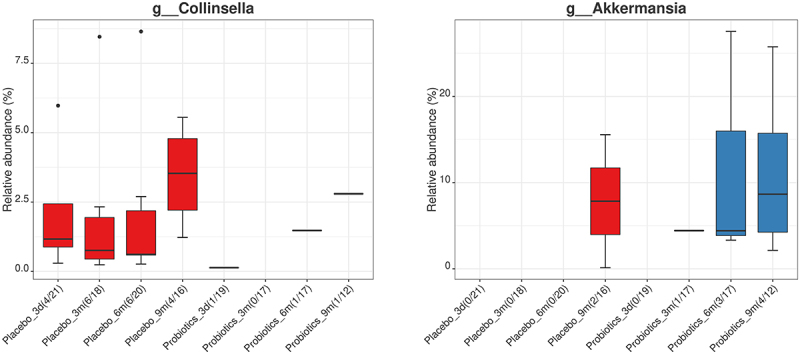
Relative abundance (%) of the genera *Collinsella* and *Akkermansia* over time (3 days [3d], 3 months [3 m], 6 months [6 m], and 9 months [9 m]) in infants from mothers treated with placebo (red) or probiotics (blue). No significant differences in differential abundance were observed between the treatment groups. However, the number of fecal samples in which the respective genera were detected, varied noticeably.

Despite the absence of differences at phylum or genus level when comparing the probiotic and placebo groups, there were, however, some interesting observations were made when considering the presence and absence of some specific genera. The genus *Collinsella* was almost consistently absent in infants from the probiotic group, as it was only detected in one infant in the probiotic group across all time points while being observed in 4–6 infants (depending on the sampling time) in the placebo group across all sampling points (Figure 7A). On the other hand, *Akkermansia* was not detected in infant GM from the placebo group before 9 months after birth, where it was detected in two fecal samples. For the probiotic group, *Akkermansia* was detected at 3, 6 and 9 months after birth, in one, three, and four fecal samples (Figure 7B).

#### Maternal antibiotic administration during delivery resulted in a decreased relative abundance of Bifidobacterium in the GM of the infants

During delivery, 18/24 mothers in the probiotic group and 14/23 mothers in the placebo group received antibiotics (Table 1). All 14 infants delivered by cesarean section (10 from the probiotic group and 4 from the placebo group) had a mother who received antibiotics during delivery, while 18 infants born at VD had a mother who received antibiotics during delivery. Administration of antibiotics during delivery was associated with a significant decrease in the relative abundance of *Bifidobacterium* (*p* = 0.006) 3 days after birth.

Administration of antibiotics during pregnancy had no effect on the GM of infants in both alpha and beta diversity or in the differential abundances of any genera to any of the sampling timepoints (3 d, 3 months, 6 months, and 9 months).

## Discussion

Probiotic therapy is increasing in popularity globally despite inconsistent clinical data and evidence to support its efficacy.^35^ We have previously shown that a probiotic intervention with Vivomixx® is feasible in pregnant women with obesity.^24^ This paper reports results regarding the follow-up visits conducted on the infants. Forty-seven infants of mothers from the primary study completed the study until 9 months after birth, indicating that the study design and intervention also is appropriate for further testing. When investigating the transferability of the probiotics included in the Vivomixx® multispecies probiotics from mothers to infants, no differential abundance between the treatment groups was observed for neither *Bifidobacterium* spp., *Lactobacillus* spp. nor *Streptococcus* spp. at any of the sampling points.

As anticipated, an increase in GM alpha over time was observed, as environmental exposures ensured colonization and thus increasing GM diversity.^7^ There was nevertheless no indication of maternal prenatal probiotic supplementation affecting infant GM diversity in the probiotic group compared with the placebo group. These findings are supported by data from other RCTs, which, despite differences in the probiotic product used and their affiliated differences in transferability from mother to child, confirmed maternal probiotic supplementation to be without influence on the alpha and beta diversity of the infant’s GMs.^36,37^ Likewise, an RCT in breastfed infants with colic randomly assigned to receiving the Vivomixx® mixture or a placebo themselves for 21 days showed no effect on the relative abundance of *Bifidobacteria* and *Lactobacilli* .^38^

Other probiotic products have shown more convincing results regarding the effects of probiotics during pregnancy on maternal and fetal GM colonization. Schultz *et al*. ^39^ showed that infants of mothers who were taking *Lactobacillus rhamnosus* strain GG (LGG) were colonized with LGG for up to 12 months after birth. Gueimonde *et al*. ^40^ showed that the administration of LGG to mothers from 2 to 4 weeks before labor and during 3 weeks after delivery changed the initial establishment of *Bifidobacteria* in newborns compared with those receiving placebo. Moore *et al*. ^41^ showed that a direct strain transfer from mothers to infants of *Bifidobacterium breve* 772058 occurred infrequently in 2/65 infants in the probiotic group and in 0/70 in the placebo group, when the probiotics were taken from 16 weeks of gestation until 3 months *postpartum*.

Besides the abundance of the Vivomixx® genera specifically, the effect of maternal prenatal probiotic administration was not visible in the differential abundance of other genera. This suggests that Vivomixx® supplementation did not lead to community-scale differences affecting the composition of other genera in the gut community. There were, however, some indications of community changes for the nearly complete depletion of *Collinsella* and enrichment of *Akkermansia* in infants from mothers of the probiotic group compared with the placebo group. A study by Gomez-Arango *et al*.^42^ found a positive correlation between *Collinsella* abundance and circulating insulin levels and that *Collinsella* abundance was increased in pregnant women with overweight or obesity when consuming a diet low on dietary fiber. Our findings suggest that supplementation of Vivomixx® may avoid at least the transfer of *Collinsella* from pregnant women with obesity to the GM of their infants.

Further investigation is needed to determine whether Vivomixx® altered the abundance of *Akkermansia* in the maternal GM and the potential effects of this. The genus *Akkermansia* comprises mucin-degrading bacteria that has been associated with beneficial metabolic activities and holds probiotic potential, since the presence of these bacteria is inversely correlated with body fat and glucose intolerance in especially murine models. Dao *et al*.,^43^ amongst others, similarly showed therapeutic potential of *Akkermansia* in terms of improving metabolic health during calorie restriction in individuals with overweight or obesity. They considered parameters such as waist-to-hip ratio, body fat distribution, insulin sensitivity, and abundance of other microbial species associated with health and found that high abundance of *Akkermansia* improved these parameters after a calorie restriction-based intervention. The slightly higher prevalence of *Akkermansia* and earlier colonization of the gut in the infants from the probiotic mother group, is therefore an interesting observation. Yet, these findings may similarly be caused by random exposure in the environment of the infants, and especially when the prevalence is this low for both treatment groups, a larger study population is needed to determine the relationship between maternal Vivomixx® supplementation and infant gut *Collinsella* and *Akkermansia* abundance. For future similar studies using Vivomixx®, preferably with more participants, it would be interesting to investigate whether this altered prevalence of *Collinsella* and *Akkermansia* could be confirmed in mothers as well as infants.

To evaluate the possible predisposition for obesity in the infants, it would have been interesting to compare the GMs of the infants to those of other infants with lean mothers, to investigate whether, *e.g.*, a difference in the *Firmicutes* to *Bacteroidetes* ratio or other differential abundances would be detectable. Since the GMs of infants from probiotic and placebo group did not differ significantly from each other in differential abundance, this comparison with microbiomes of lean women may reveal whether these infants’ GMs are relatively like a ‘lean microbiome’ or significantly altered by the maternal obesity. Maternal prenatal Vivomixx® supplementation did not result in differences in prevalence of any diseases, including atopic dermatitis, colic, or allergy. Other studies have found diminished disease prevalence, severity, or duration for diseases such as allergy and colic, when administering probiotics.^38,44^ It is likely that the result will differ according to the probiotic product administered.^23^ For instance, although the literature is not in complete agreement, some studies have suggested that allergic infants have a decreased abundance of *Bifidobacteria* and *Bacteroides* compared with non-allergic infants.^44^ As Vivomixx® contains three *Bifidobacteria* spp. and no *Bacteroides* spp., other probiotic products may be more efficient in relation to prevent allergic diseases. Also, the period in which the probiotic is being administered can affect the results, as prolonged exposure may improve probiotic engraftment into the gut. Several of the studies reporting improved effect of probiotics on disease prevalence administered probiotics during pregnancy and after birth^44^ or directly to the infants,^38^ whereas we did not continue probiotic supplementation beyond the time of birth. A considerable number of studies in allergy prevention using probiotics have had success with a combination of prenatally and postnatally (to the mother and/or infant) administered probiotics, whereas studies using prenatal or postnatal supplementation exclusively, have failed to obtain similar results.^44^

One of the great challenges of using probiotics as a GM modulator is that the probiotics merely appear to have a short-term effect, as the abundance of the probiotics quickly diminishes, when the probiotics are no longer supplemented.^45^ This is likely a result of the dense colonization and high degree of competition amongst commensal bacteria, making permanent engraftment into the gut challenging. However, promising results have been observed for lasting disease-preventing effects, when probiotics were given before the GM reached an established community structure, such as in infants. A Norwegian study by Dotterud *et al*. ^46^ found that probiotics, including *Lactocaseibacillus rhamnosus* GG, *L. acidophilus* and *Bifidobacterium animalis* subsp. *Lactis* administered maternally from 36 weeks of gestation until 3 months postnatally, reduced the incidence of atopic dermatitis in the infants with 40%. Another study, investigating the effect of Vivomixx® multistrain probiotic in infants suffering from colic, found that this probiotic reduced the daily number of minutes of inconsolable crying.^38^

The initial colonization of the gut happens during early life and plays a vital part in the health and development of the infant as well as for the risk of medical conditions later in life. For instance, exposure to antibiotics early in life has been associated with an increased risk of developing diseases such as obesity, type 1 and 2 diabetes, inflammatory bowel diseases, allergy, and asthma later in life.^47^ However, it is important to keep in mind that due to the differences in the underlying mechanisms of various probiotic strains results found for one probiotic product or strain may not be applicable to other products or strains.^23^

## Strengths and limitations

Our study has some limitations related to the sample size since the study was a feasibility study. In addition, the included groups varied regarding the different proportion of vaginal deliveries and cesarean section in the probiotic and placebo groups. The strong degree of random fluctuations of the infant GM caused by a varying degree of environmental exposures^34^ complicated the analysis, as it might blur the potential treatment-associated effects on the GM. This can be facilitated by more observations, but also an improvement of the methodic differences complicating the analysis. For instance, the sensitivity of DESeq2 for rare observations may not be ideal for the analysis of the highly individual and unbalanced composition of infant gut microbiomes, while the Wilcoxon rank-sum test may be too insensitive. Future methodic consensus may shed a light over which findings to consider the most accurate or at least ease comparison across studies. In addition, there has been a lot of development on microbiome analyzes since we analyzed these samples with the 16S method. We recommend future studies to use state-of-the-art methods like metagenomic sequencing (MGS), including the application of multi-omics characterization for the microbiome analyzes.

Furthermore, the prevalence of disease (including colic and allergy) was overall very low, and an increased number of participants are needed to eliminate the effect of randomness. Inclusion of participants predisposed to any of these diseases may also render the results regarding finding an effect or not.

To confirm the observations of this study, a follow-up later in the life of the infants could be performed to report potential differential disease development between the infants of the two treatment groups. Unfortunately, our study was only planned with 9-month follow-up after delivery. Likewise, further studies should focus on direct supplementation of probiotics to the infant to increase long-term richness, indicating the necessity of postnatal probiotic administration, either indirectly to the mother or directly to the infant, to induce detectable changes in the GM composition. In addition, dietary intake is known to be a driver of microbiome variation, therefore comprehensive collection of dietary intake data from boththe women (during pregnancy or while breastfeeding) andthe infants could have been appropriate for microbiome analyzes adjustments. Especially, an estimation of prebiotics intake could have been relevant since prebiotics stimulate the growth of, e.g., *Bifidobacteria* and *Lactobacilli* and thereby also impact the gut microbiome composition.

## Supplementary Material

Supplemental Material

## Data Availability

Due to the nature of this research, participants of this study did not agree for their data to be shared publicly, so supporting data is not available. The data are available from the corresponding author, AMP, upon reasonable request.
