# Supplementary material for: Multi-strain probiotics during pregnancy in women with obesity influence infant gut microbiome development: results from a randomized, double-blind placebo-controlled study
Source: Gut Microbes. 2024 Apr 9;16(1):2337968. doi: 10.1080/19490976.2024.2337968 (PMC11005804; doi:10.1080/19490976.2024.2337968)
Supplement: Supplemental Material [file KGMI_A_2337968_SM9582.zip › KGMI_A_2337968_SM/Supplemenatry tables.docx]

**Supplementary tables**

**Table S1.** Number of fecal samples from the infants contributing to each treatment group (mothers treated with probiotics or placebo during pregnancy) and sampling timepoint (three days, three months, six months, and nine months).

|  | **3 days** | **3 months** | **6 months** | **9 months** | **Total** |
| --- | --- | --- | --- | --- | --- |
| **Probiotics** | 19 | 17 | 17 | 12 | 65 |
| **Placebo** | 21 | 18 | 20 | 16 | 75 |
| **Total** | 40 | 35 | 37 | 28 | 140 |

**Table S2**. Alpha diversity of the infant GM in the group of mothers treated with probiotics during pregnancy identified at each timepoint after birth (three days [3d], three months [3m], six months [6m] and nine months [9m]). A significant increase (*p* < 0.05) in alpha diversity over time for the probiotic treatment group was generally observed for all alpha diversity metrics (Richness, Shannon, Simpson, and Evenness). Considering the baseline and final sampling times, there was a significant difference (*p* < 0.001) between three days and nine months after birth for all alpha diversity metrics. However, there were generally no significant differences between three months and six months after birth, and this also applied to some of the other adjacent sampling times; *e.g.,* six months and nine months after birth, depending on the diversity metric considered. This was tested using Wilcoxon rank sum test (*p* value adjustment method: Benjamini & Hochberg).

| **Probiotic: Alpha diversity** (*p* value) | **3d vs. 3m** | **3d vs. 6m** | **3d vs. 9m** | **3m vs. 6m** | **3m vs. 9m** | **6m vs. 9m** |
| --- | --- | --- | --- | --- | --- | --- |
| Richness | 0.002 | <0.001 | <0.001 | 0.050 | 0.008 | 0.147 |
| Normalized Richness | <0.001 | <0.001 | <0.001 | 0.082 | 0.002 | 0.033 |
| Effective Richness | <0.001 | <0.001 | <0.001 | 0.276 | 0.008 | 0.009 |
| Shannon Index | 0.003 | <0.001 | <0.001 | 0.508 | 0.063 | 0.150 |
| Shannon Effective | 0.004 | <0.001 | <0.001 | 0.514 | 0.063 | 0.150 |
| Simpson Index | 0.011 | 0.001 | <0.001 | 0.604 | 0.230 | 0.471 |
| Simpson Effective | 0.015 | 0.003 | 0.001 | 0.598 | 0.230 | 0.448 |
| Evenness | 0.011 | 0.005 | <0.001 | 1.000 | 0.174 | 0.174 |

**Table S3.** Alpha diversity of the infant GM in the group of mothers treated with placebo during pregnancy identified at each timepoint after birth (three days [3d], three months [3m], six months [6m] and nine months [9m]). A significant increase (*p* < 0.05) in alpha diversity over time for the placebo treatment group was generally observed for all alpha diversity metrics (Richness, Shannon, Simpson, and Evenness). Considering the baseline and final sampling times, there was a significant difference (*p* < 0.01) between three days and nine months after birth for all alpha diversity metrics. However, there were generally no significant differences between six months and nine months after birth, and this also applied to some of the other adjacent sampling times; *e.g.,* three days and three months after birth, depending on the diversity metric considered. This was tested using Wilcoxon rank-sum test (*p* value adjustment method: Benjamini & Hochberg).

| **Placebo: Alpha diversity** (*p* value) | **3d vs. 3m** | **3d vs. 6m** | **3d vs. 9m** | **3m vs. 6m** | **3m vs. 9m** | **6m vs. 9m** |
| --- | --- | --- | --- | --- | --- | --- |
| Richness | 0.002 | <0.001 | <0.001 | 0.017 | 0.001 | 0.124 |
| Normalized Richness | <0.001 | <0.001 | <0.001 | 0.034 | <0.001 | 0.014 |
| Effective Richness | 0.002 | <0.001 | <0.001 | 0.025 | <0.001 | 0.043 |
| Shannon Index | 0.094 | <0.001 | <0.001 | 0.005 | <0.001 | 0.190 |
| Shannon Effective | 0.0919 | <0.001 | <0.001 | 0.005 | 0.001 | 0.188 |
| Simpson Index | 0.289 | 0.002 | 0.001 | 0.005 | 0.004 | 0.583 |
| Simpson Effective | 0.283 | 0.003 | 0.001 | 0.007 | 0.005 | 0.583 |
| Evenness | 0.408 | 0.013 | 0.005 | 0.011 | 0.005 | 0.407 |

**Table S4.** Comparison of alpha diversity of the infant GM compared between infants from mothers treated with probiotics or placebo during pregnancy at each time point. No significant difference (*p* > 0.05) in alpha diversity was observed between the two groups (probiotics and placebo) at any of the time points (three days [3d], three months [3m], six months [6m], and nine months [9m]) for any of the alpha diversity metrics (Richness, Shannon, Simpson and Evenness). This was tested using Wilcoxon rank-sum test (*p* value adjustment method: Benjamini & Hochberg).

| **Probiotic vs. Placebo:**  **Alpha-diversity** (*p* value) | **3 days** | **3 months** | **6 months** | **9 months** |
| --- | --- | --- | --- | --- |
| Richness | 0.375 | 0.440 | 0.705 | 0.889 |
| Normalized Richness | 0.472 | 0.558 | 0.626 | 0.926 |
| Effective Richness | 0.701 | 0.332 | 1.000 | 0.999 |
| Shannon Index | 1.000 | 0.179 | 0.667 | 1.000 |
| Shannon Effective | 1.000 | 0.179 | 0.651 | 0.999 |
| Simpson Index | 0.893 | 0.271 | 0.624 | 0.680 |
| Simpson Effective | 0.903 | 0.271 | 0.619 | 0.680 |
| Evenness | 0.917 | 0.145 | 0.579 | 0.943 |
|  |  |  |  |  |
|  |  |  |  |  |
