# Supplementary material for: Multi-strain probiotics during pregnancy in women with obesity influence infant gut microbiome development: results from a randomized, double-blind placebo-controlled study
Source: Gut Microbes. 2024 Apr 9;16(1):2337968. doi: 10.1080/19490976.2024.2337968 (PMC11005804; doi:10.1080/19490976.2024.2337968)
Supplement: Supplemental Material [file KGMI_A_2337968_SM9582.zip › KGMI_A_2337968_SM/Supplementary figure captions.docx]

Fig. S1. Alpha diversity according to probiotic (blue) or placebo (red) treatment plotted using different alpha diversity metrics, including Richness, Evenness, Shannon Index and Simpson Effective. No significant differences were observed between infants from mothers treated with placebo (red) or probiotics (blue) at any of the sampling times nor for any of the diversity metrics.

Fig. S2. Principal coordinate analysis (PCoA) plot of the beta diversity (Bray-Curtis dissimilarity) according to the mothers probiotic or placebo treatment during pregnancy at each sampling time (three days [3d], three months [3m], six months [6m], and nine months [9m], respectively). No significant differences were observed.

Fig. S3. Principal coordinate analysis (PCoA) plot of the beta diversity for all time points according to infants from mothers treated with placebo or probiotics during pregnancy (weighted UniFrac). No significant differences were found between the probiotic and placebo groups to any of the timepoints; however, there was a significant over-time difference for both groups when comparing beta diversity at the different time points (three days [3d], three months [3m], six months [6m], and nine months [9m], respectively).

Fig. S4. Phylum-level differential abundance according to infants from mothers treated with placebo or probiotics over time. No significant differences were observed between the probiotic and placebo treatment for any of the time points (three days [3d], three months [3m], six months [6m], and nine months [9m], respectively).

Fig. S5. Effect of delivery mode on alpha diversity (Shannon Effective). Shannon Effective significantly increased over time for both delivery modes. Vaginally born children (Vaginal) has a significantly higher Shannon Effective compared with infants delivered by cesarean section (Sectio) three days (3d) after birth (*p* = 0.01). For the remaining sampling times (three [3m], six [6m], and nine [9m] months), no significant differences in Shannon Effective between the two delivery modes were found.

Fig. S6. Effect of delivery mode on beta diversity (Bray-Curtis dissimilarity) three days after birth: Beta diversity significantly differed when comparing vaginally delivered infants (Vaginal) to infants delivered by cesarean section (Sectio) (*p* = 0.008).
