# Supplementary figures and images for: Multi-strain probiotics during pregnancy in women with obesity influence infant gut microbiome development: results from a randomized, double-blind placebo-controlled study
Source: Gut Microbes. 2024 Apr 9;16(1):2337968. doi: 10.1080/19490976.2024.2337968 (PMC11005804; doi:10.1080/19490976.2024.2337968)

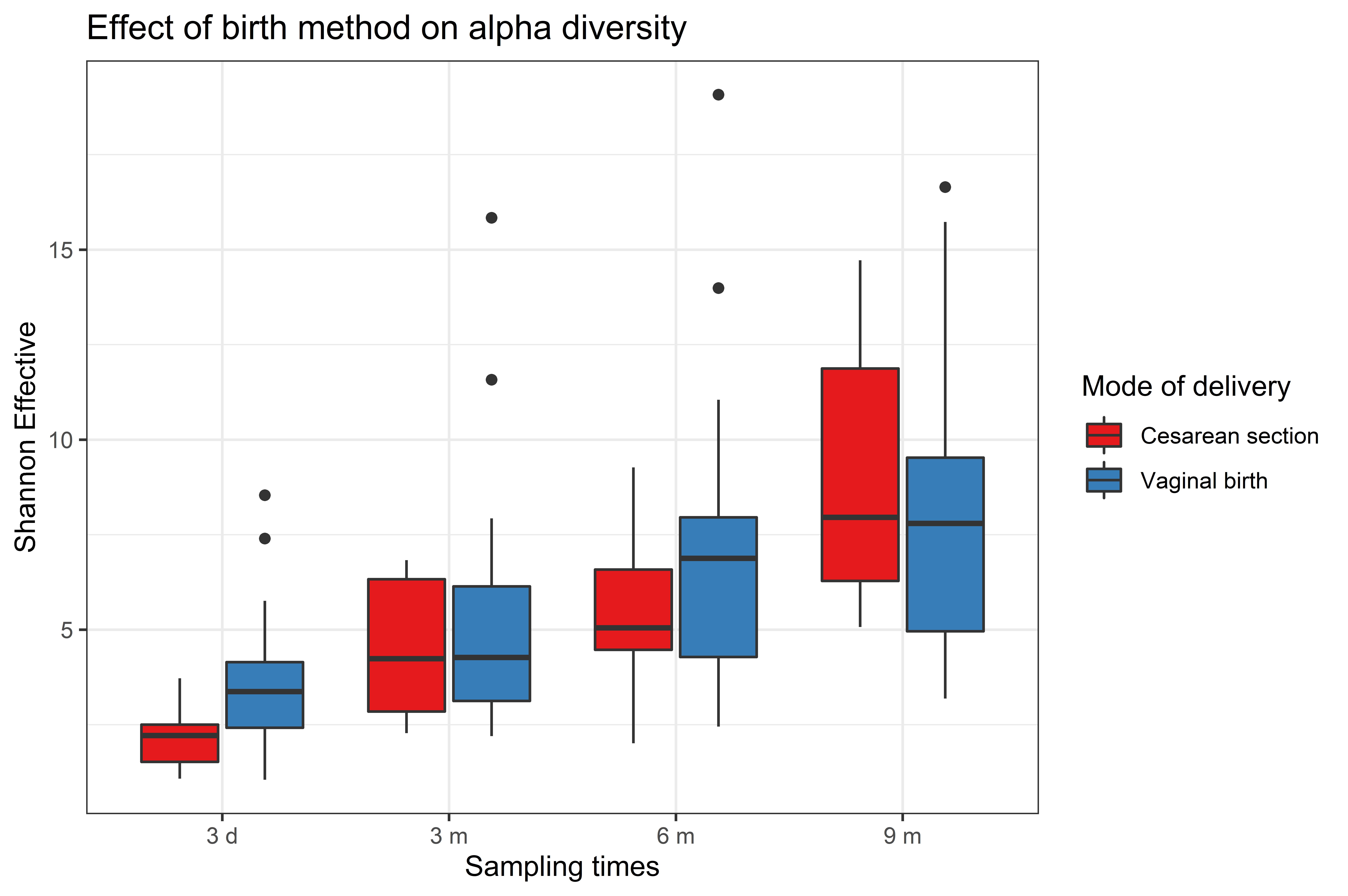

Supplement: Supplemental Material [file KGMI_A_2337968_SM9582.zip › KGMI_A_2337968_SM/Supplementary Figure 5.jpg]
